# Carbolic Acid Poisoning*Read before the West Texas Medical Association, July, 1899.

**Published:** 1899-09

**Authors:** Frank Paschal

**Affiliations:** City Health Officer, San Antonio, Texas


### TEXAS MEDICAL JOURNAL.

ESTABLISHED JULY, 1885.

PUBLISHED MONTHLY.—SUBSCRIPTION $1.00 A YEAR.

VOL. XV. AUSTIN, SEPTEMBER, 1899. No. 3.

### Original Contributions.

For the Texas Medical Journal.

### Carbolic Acid Poisoning.*

BY FRANK PASCHAL, M. D., CITY HEALTH OFFICER, SAN ANTONIO, TEXAS.

The use of carbolic acid as a disinfectant and germicide makes it a common article in households, and cases of poisoning, intentional and accidental, by it are of not infrequent occurrence. The fatal dose seems uncertain. It is said that it will be a remarkable exception when two tablespoonfuls of the deliquesced acid fails to kill, and that a tablespoonful will be more likely than not to prove fatal. A case is reported, however, where three ounces of pure acid was swallowed for suicidal purposes by a female 19 years old, and the case recovered. This contradicts the assertion that the mortal minimum dose is an ounce and two-thirds. An amount which would prove fatal in one person will not do so in another, much depending, no doubt, on individual susceptibility, the condition of the stomach at the time of taking it and also upon the promptness and efficiency of treatment. The rapidity of the toxic effect of carbolic acid is only excelled by hydrocyanic acid. Cases are on record where persons have died in a few minutes after swallowing it. One

Read before the West Texas Medical Association, July, 1899.

case where death resulted in two or three minutes, the patient dying in rapid coma.

When taken and absorbed in massive doses it acts as a depressor upon the *medulla oblongata,* spinal cord, and brain centres, giving rise to loss of sensibility and reflexes, stertorous breathing and coma. The following case has been of interest and instructive to me, and I trust may prove the same to you.

At 2:30 a. m., May 20, 1899, I received a telephone message to go to the City Hall to see a woman who had taken carbolic acid. I directed that she be sent to the hospital, and learned a few moments afterwards that she was not at the hall, but an inmate of a house on the west side of the San Pedro creek. I got telephone connection with the house, and directed that she be given a quantity of sweet oil. They succeeded in giving her about four ounces, when she ran out of the house into the yard and fell in a state of unconsciousness, it being then about ten minutes from the time of taking the acid. I arrived at the hospital about 2:55, and found her in the female ward. She was a little woman, 23 years old, weighed about 120 pounds. She had taken an ounce of pure carbolic acid. She was unconscious, pale, skin bathed in perspiration, surfaces cold and clammy, reflexes entirely abolished, breathing stertorous, rapid, not less than 80 to 100 a minute, loud tracheal rales. Pulse rapid, irregular in action and volume, at times full, tumultuous and intermittent, and again feeble and exceedingly rapid, and then again ceasing for a few seconds to beat at all, and then off, so to speak, at a tangent.

The pupils were neither dilated nor contracted, but were insusceptible to light, and there was absence of corneal and conjunctival reflex. I doubted her living long enough to even prepare for treatment. A hypodermic injection of 1-20 strychnine was given at 3 a. m. The stomach was washed at the same hour with a large quantity of soapsuds water, the washing being continued until no further odor of acid could be detected, when one pint of sweet oil was thrown into the stomach, and this was followed by a quart or more of warm soapsuds with a quantity of bicarbonate of soda, and after withdrawing the oil and water a pint of oil was thrown into the stomach and left there.

3:15, 1-40 gr. of strychnine hypodermically.

3:45, 1-20 gr. of strychnine hypodermically.

4:30, 1-50 gr. of nitro-glycerine and 20 drops of tinct. dig. hypodermically.

I left her at 5 a. m. unconscious, and but little change in general condition, and expected her to die in a few minutes, and ordered strychnine, nitro-glycerine and digitalis to be repeated at short intervals.

5 a. m., strychnine, 1-20.

6 a. m., patient became conscious and vomited frequently. She was very restless and depressed, the bowels moving frequently a yellow-greenish fluid. Passed six ounces of urine, which was not collected.

9 a. m. found her perfectly conscious, pulse 80, regular and of good volume. She was suffering excruciating pains in both renal regions. I gave a pint of normal salt solution under the left breast, but she fought so strenuously against repeating it under the right one that I had to desist and gave enema of normal salt solution, which, however, was not retained. Hot fomentations of strong infusions of digitalis leaves were constantly applied over the kidneys. Thirst intense, and her pleadings for water could not be withstood, and she was allowed all of the cold water she wanted.

12 m., still very restless, vomiting yellow fluid, bowels moving same character of fluid. Hypodermic strychnine, 1-40 gr., ordered every four hours.

6 p. m., enema salt solution every two hours; not able to retain them.

May 21st, 6 a. m., very restless night, slept about two hours. Vomited freely and frequently, bowels moved four times.

9 a. m., one-half gr. morphine hypodermically.

12 m., resting easy, has not vomited since 9:30. Bowels moved slightly.

1 p. m., four ounces of peptonized milk given by rectum every four hours. Retained the greater part.

5 p. m., rested quite easy all day since morphine—ordered one-fourth gr. morphine hypodermically every four hours. Gave diluted milk by mouth.

11:30 p. m., passed four ounces urine. Rested well until this hour, choking spell. Painful micturition last night.

May 22nd, 6 a. m., from 11:30 had little rest, suffering with pain in bladder and in region of ovaries. Dysphagia. Expectorating good deal of muco-pus from larynx and bronchi. Has not vomited. Nourishing enemas retained until 3 a. m. Watery movements with whitish flakes. Passed urine every two to three hours during the night, amounting to 10 or 12 ounces. Nourishing enemas every two hours. Two ounces at a time retained in this way.

3 p. m., continues to improve. Vomiting ceased, able to take a little diluted milk by mouth.

May 23rd, 6 a. m., rested better during the night. Strychnine 1-40 gr. every six hours. Morphine every four hours, ¼ gr. Menstruating freely. Had gotten over her menstrual period eight days ago, now returned on her. Abdomen painful and tympanitic. Hot digitalis fomentations applied to the abdomen and discontinued from over the kidneys.

6 p. m., feeling better and abdominal distension and pain relieved. Applications of digitalis discontinued.

May 24th, 6 a. m., slept well; passed urine twice; amount about 8 ounces. Bowels moved once.

6 p. m., found her crying for food; ordered a soft-boiled egg, which she enjoyed and retained. Milk was given frequently, peptonized, by mouth.

May 25th, 6 a. m., still improving; slept well; passed 8 ounces of urine during the night, and is taking milk toast, soft egg, milk, strychnine, 1-40 gr., every six hours, hypodermically. Morphine, ¼ gr., at about the same intervals. Menstrual flow ceased. Does not suffer much pain in swallowing, and none now in the stomach. I consider her in a fair way to recovery.

In conclusion, I will say that she was accustomed to taking morphine, and I therefore found it necessary to continue its use. I found only a little albumen in the urine, and the urine never bloody or much changed in color. I think it unwise not to allow patients in such cases to have all of the water they want, and it is cruel to withhold it. The thirst was so intense that during a minute's absence of the nurse from the bedside she got up and went to the hydrant and drank freely of water.

Now, it is the danger of death from immediate paralysis of the nerve centers that we are to fear in these cases, as the caustic or escharatic effect upon the mucous membrane of the stomach and bowels is of secondary import, and we must guard against the paralysis of brain and cord. This woman was so profoundly unconscious that any surgical operation could have been performed without the slightest sense of pain. Passing the stomach tube did not excite the least reflex.

The treatment to be effectual must be prompt, and as the case has recovered on the line of treatment that was followed, I have nothing more to suggest, except to say that to wait on the soluble sulphates to act and form sulpho-carbolates and to trust to emetics to clear out the stomach, whether given as apo-morphia or by mouth, is only to waste time and probably lose life. Strychnine should be used heroically, as the fight should be made to get the ascendency over the depressing effect of the acid on the cerebro-spinal centers.

